# Metformin disrupts malignant behavior of oral squamous cell carcinoma via a novel signaling involving Late SV40 factor/Aurora-A

**DOI:** 10.1038/s41598-017-01353-8

**Published:** 2017-05-02

**Authors:** Chang-Han Chen, Hsin-Ting Tsai, Hui-Ching Chuang, Li-Yen Shiu, Li-Jen Su, Tai-Jan Chiu, Sheng-Dean Luo, Fu-Min Fang, Chao-Cheng Huang, Chih-Yen Chien

**Affiliations:** 1 Institute for Translational Research in Biomedicine, Kaohsiung Chang Gung Memorial Hospital, Kaohsiung, Taiwan; 2 Department of Applied Chemistry, and Graduate Institute of Biomedicine and Biomedical Technology, National Chi Nan University, Nantou, Taiwan; 3 Center for Infectious Disease and Cancer Research, Kaohsiung Medical University, Kaohsiung, Taiwan; 4 Department of Otolaryngology, Kaohsiung Chang Gung Memorial Hospital, and Chang Gung University College of Medicine, Kaohsiung, Taiwan; 5 Department of Medical Research, E-Da Hospital, I-SHOW University, Kaohsiung, Taiwan; 6 Cell Therapy and Research Center, Department of Medical Research, E-Da Cancer Hospital, Kaohsiung, Taiwan; 7 Graduate Institute of Systems Biology and Bioinformatics, National Central University, Jhongli, Taiwan; 8 Departments of Hematology-Oncology, Chang Gung University College of Medicine, Kaohsiung, Taiwan; 9 Department of Radiation Oncology, Kaohsiung Chang Gung Memorial Hospital and Chang Gung University College of Medicine, Kaohsiung, Taiwan; 10 Department of Pathology, Kaohsiung Chang Gung Memorial Hospital and Chang Gung University College of Medicine, Kaohsiung, Taiwan

## Abstract

Conventional therapeutic processes in patient with OSCC are associated with several unfavorable effects leading to patients with poor survival rate. Metformin has been shown to protect against a variety of specific diseases, including cancer. However, the precise roles and mechanisms underlying the therapeutic effects of metformin on OSCC remain elusive. In the current study, *in vitro* and xenograft model experiments revealed that metformin inhibited growth and metastasis of oral cancer cells. Importantly, metformin-restrained tumorigenesis of oral cancer was accompanied with strong decrease of both Aurora-A and Late SV40 Factor (LSF) expressions. Furthermore, LSF contributed to Aurora-A-elicited malignancy behaviors of oral cancer via binding to the promoter region of Aurora-A. A significant correlation was observed between LSF and Aurora-A levels in a cohort of specimens of oral cancer. These findings showed that a novel LSF/Aurora-A-signaling inhibition supports the rationale of using metformin as potential OSCC therapeutics.

## Introduction

Oral squamous cell carcinoma (OSCC) is a common malignancy in South-East Asia and India. It is believed to be related to smoking, alcohol consumption, betel nut chewing, and certain viral infections. Betel nut chewing constitutes a great threat to public health in Taiwan, especially as it affects the occurrence of oral cancer. In Taiwan, it is estimated that more than 5400 persons were diagnosed with oral cancer and more than 1800 persons died of this disease in 2013. Despite the recent advances in technology and multidisciplinary intervention, only modest improvements in the survival of oral cancer have been achieved and these are attributed mainly to diagnosis at an early stage, rather than to therapeutic interventions^1^. This means that standard treatment fails in a significant proportion of patients and salvage surgery is unsatisfactory, although it depends on the stage of the recurrent tumor^2^. Therefore, it is essential to develop a new therapeutic strategy for treating these advanced tumors.

Metformin is an antihyperglycemic agent commonly used to treat patients with type 2 diabetes mellitus (DM). It decreases hyperglycemia by suppressing hepatic gluconeogenesis^3^. Epidemiological studies show that patients with DM are at increased risk of breast cancer and hepatocellular carcinoma^4, 5^. However, some groups of patients with DM and breast cancer or hepatocellular carcinoma, especially those taking metformin for blood sugar control, show better survival^4, 6^. It was estimated that the risk of hepatocellular carcinoma is reduced by 70%^4^, while a higher pathological complete response rate is achieved in breast cancer^6^. Among the head and neck cancer, those patients who took metformin for DM control would show a better overall survival and disease free survival in laryngeal cancer^7^. These clinical results have prompted interest in further evaluating the role of metformin in cancer treatment.

A growing body of evidence have demonstrated that metformin significantly inhibits the tumor growth of many cancer cells, such as breast, prostate and gastric cancer, and lymphoma *in vitro* and *in vivo* ^8^. The well-known mechanism underlying the anti-cancer effect of metformin is adenosine monophosphate (AMP)-activated protein kinase (AMPK) signaling. AMPK, a serine/threonine protein kinase, is a cellular energy sensor, which is activated in response to an increased AMP/adenosine triphosphate (ATP) ratio caused by various cellular stresses. The AMPK-dependent pathway requires activation of the upstream liver kinase B1 (LKB1). However, recent *in vitro* experiments reported that metformin may be through AMPK-independent mechanisms to suppress tumor growth^9^. These studies point out that metformin may evoke a variety of signaling to prevent cancer development.

The transcription factor LSF (Late SV40 Factor), also assigned as TFCP2, encodes a 502 amino acids with a predicted molecular weight of 57 kDa and is involved in many biological events, including in cell cycle regulation, DNA synthesis, cell growth and Alzheimer disease^10^. LSF could be a hub target of a network of proteins, involving osteopontin, c-Met, and MMP-9 to regulate tumor progression, angiogenesis and metastasis in human cancers^11–13^. Aberrant expression of LSF was found in HCC. In addition, the level of LSF expression displays a positively correlation with the stage and grade of the tumor, suggesting that LSF expression promotes the tumor towards a more aggressive phenotype^14^. Conversely, LSF plays a tumor suppressor role in melanoma through increasing p21 expression. These contradictory results indicated that the functional role of LSF in human cancers is diverse. However, there is little evidence to suggest a potential role for LSF in OSCC. In addition, the effect of metformin to LSF expression in oral cancer is still unclear.

Aurora-A, also named STK6, located on chromosome 20q13, contains 403 amino acid and has a molecular mass of 46 kDa. In normal tissues or cells, Aurora-A expression level is controlled via APC/C-Cdh1-dependent and proteasome-mediated proteolysis pathways^15^. In human cancers, Aurora-A is overexpressed or amplification in a variety of tumors and its expression also significantly associated with poor disease-free or overall survival of patients, including OSCC^16, 17^, suggesting that Aurora-A may represent a promising prognostic biomarker. In the last decade, several Aurora-A inhibitors have been developed and tested in clinical trials for their efficacy in human cancers. Several studies have emphasized the incremental therapeutic efficacy and suppressed tumor progression when Aurora-A inhibitor combining with conventional chemotherapeutic drugs^15^. These results indicated that Aurora-A displays a decisive role in human cancer development. However, the detailed role acted by aberrant Aurora-A signaling in OSCC has not been illustrated. Moreover, the relationship between Aurora-A and LSF in human OSCC is unknown.

In this current study, we investigated the therapeutic potential of metformin in oral cancer cells and in the tumor-bearing xenograft model. We also explored a critical role of Aurora-A in regulation of metformin sensitivity. Metformin suppressed cell growth and metastasis by inhibition of Aurora-A expression *in vitro* and *in vivo*. Furthermore, we identified LSF as a novel upstream mediator of Aurora-A signaling and showed that LSF mediated Aurora-A-induced carcinogenesis in oral cancer. Importantly, the Aurora-A expression level was highly positive correlated with LSF expression in oral cancer specimens. Taken together, we provided evidence that metformin suppressed cancer development in part through LSF/Aurora-A signaling in oral cancer.

## Results

### Metformin prevents the growth and colonigenic potential of oral cancer cells

**Figure 1:**
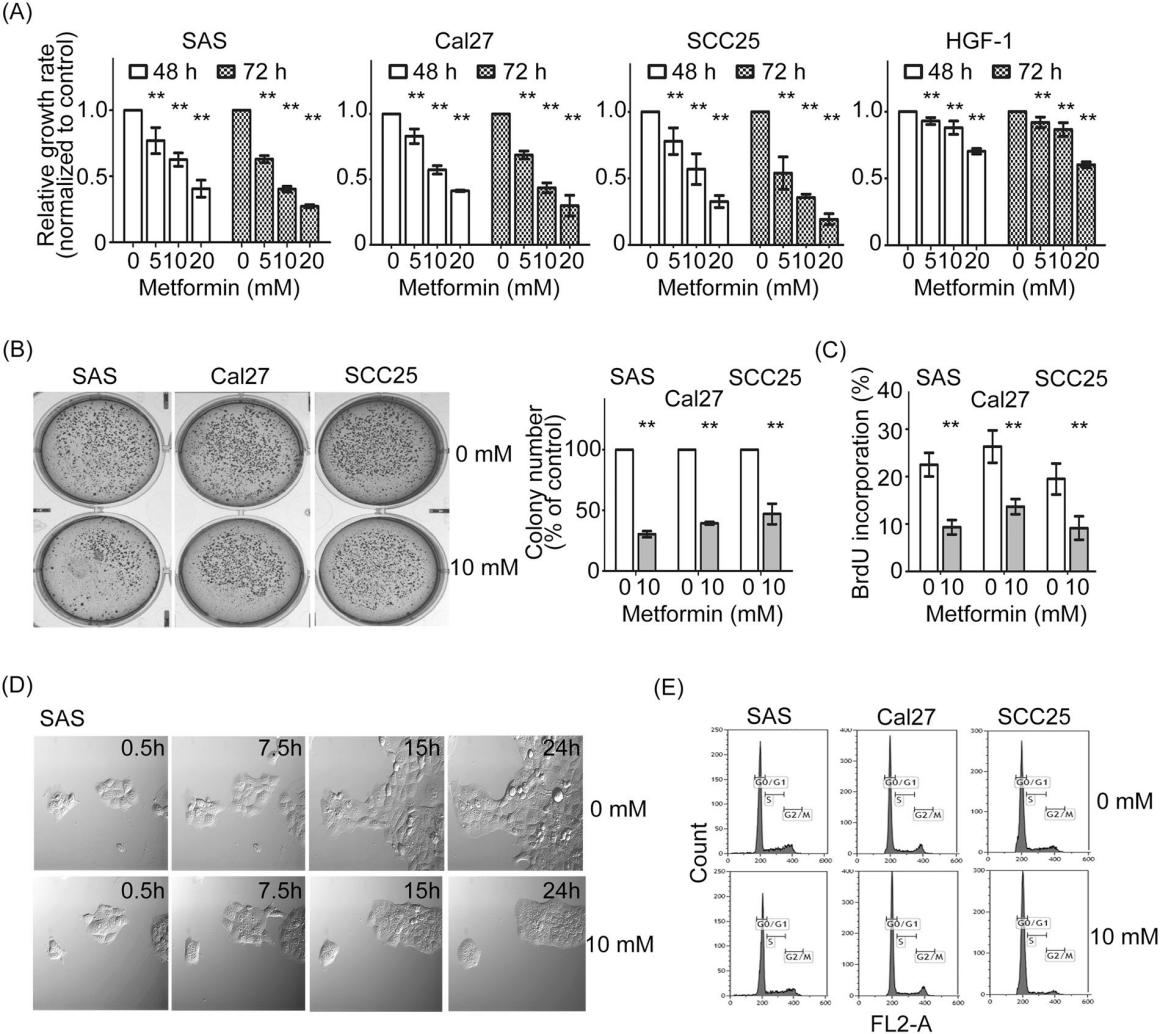
Treatment with metformin reduces the viability of oral cancer cells. (**A**) Proliferation assays performed with SAS, Cal27, SCC25, and HGF-1 cells show cell viability in the presence of metformin. (**B**) The effect of metformin on colony formation in SAS, Cal27, and SCC25 cells. Representative images and colony numbers were shown. (**C**) Forty-eight hours later, cells were subjected to BrdU incorporation assays. (**D**) Effect of metformin on progression in SAS cells. Asynchronously SAS cells were treated with 10 mM metformin and then subjected to *in vivo* time-lapse microscopy. (**E**) After treatment with metformin (10 mM) for 48 h, SAS, Cal27, and SCC25 cells were examined using PI staining. The cell cycle distribution was measured by flow cytometry. Data are represented as mean ± SD of three individual experiments. (**p < 0.01).

To address whether metformin can affect oral cancer cell growth, SAS, Cal27 and SCC25 oral cancer cell lines as well as normal gingival cell line HGF-1 cells were used to evaluate the metformin regulation of cell growth. As shown in Fig. 1A, SAS, Cal27 and SCC25 cells with metformin treatment had a significant decrease in cell growth at a dose and time-dependent manner. However, HGF-1 cells were resistant to metformin treatment up to 10 mM. These results indicate that metformin specifically restrains oral cancer cell growth, whereas normal gingival cell line are tolerant to metformin. Consistent with the cell viability assays, the clonogenic ability and BrdU incorporation were decreased by approximately 50–70% and 50% respectively, after treatment with 10 mM metformin compare to control group (Fig. 1B and C). Furthermore, the influence of metformin was further verified by time-lapse microscopy using SAS cells treated with 10 mM of metformin. Figure 1D and Supplementary Information 1, indicated that cells exposed to metformin showing delay in growth. To evaluate if the inhibition of oral cancer cell growth by metformin was mediated through cell cycle arrest, FACS analysis of DNA content was performed on 3 cell lines treated with increasing doses of metformin (5 to 10 mM) for 48 hours. FACScan analysis showed that the distribution of cell cycle of oral cancer cells treated with metformin was not alteration, compared to control group (Fig. 1E). Altogether, metformin could efficiently prevent the ability of cell growth, but not influence cell cycle distribution.

### Metformin inhibits the abilities of migration and invasion of oral cancer cells *in vitro*

**Figure 2:**
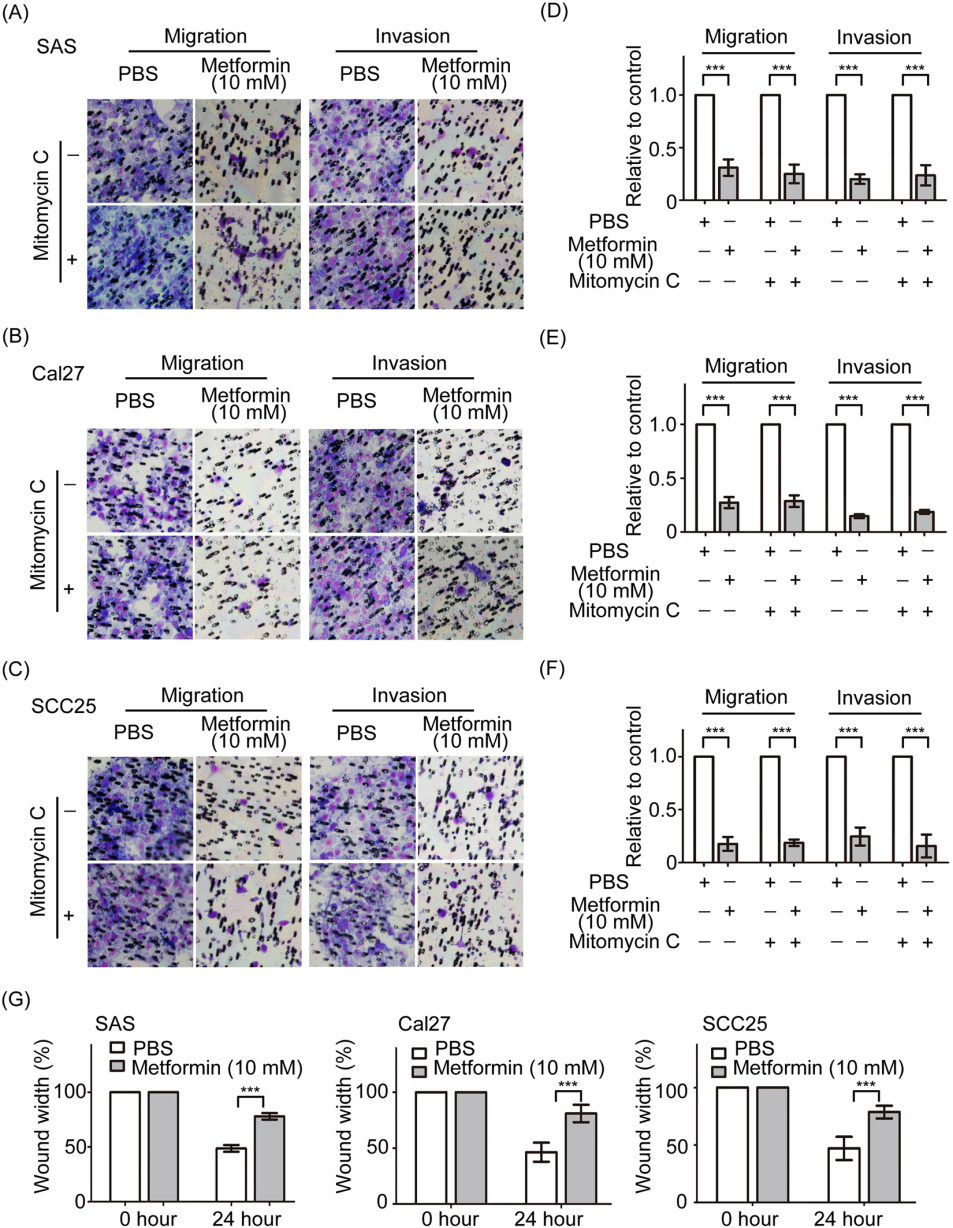
Metformin prevents the migratory and invasive abilities of oral cancer cells. Metformin induced cell migration and invasion in the absence and presence of mitomcycin C in SAS (**A**) Cal27 (**B**) and SCC25 (**C**). Cells were pretreated with or without 10 mg/ml mitomycin C for 2 h and were then plated onto the top of Transwell inserts with or without Matrigel. The migrated or invaded cells to the surface of lower membrane were counted. (**D**–**F**) The migrated or invaded cells were quantitatively analyzed. Columns are the average of three independent experiments. (**G**) SAS, Cal27 and SCC25 cells were treated with metformin and then were administered the wound-healing assay. Bar graphs show the percentage of wound closure at 24 h after scratching. Data are represented as mean ± SD of three individual experiments. (***p < 0.001).

Patients with metformin treatment had better survival chance in head and neck cancer noted from other reports. It suggests that metformin could suppress the invasive behavior of oral cancer cells. To address this hypothesis, the Transwell assay was performed to determine the migratory and invasive abilities of oral cancer cells after metformin manipulation. To exclude the effect of cell proliferation on cell migration and invasion assay, cells were treated with mitomycin C to arrest cell proliferation. As shown in Fig. 2A–C, metformin dramatically suppressed migratory and invasive abilities of SAS, Cal27 and SCC25 cells in the absence or presence of mitomycin C. Quantitatively speaking, metformin constrained SAS, Cal27 and SCC25 cell migration at a rate that were about 6.5–8.2-fold, 6.4–8.2-fold, and 7.6–8-fold low than in the control groups. Moreover, the invasive ability of SAS, Cal27 and SCC25 cells treated with metformin were approximately 7–8-fold, 7.5–8.5-fold and 7.8–8.8-fold low than that of the controls (Fig. 2D–F). Furthermore, the wound-healing assay was performed to confirm the inhibitory effect of metformin on oral cancer cell migration (Fig. 2G). Taken together, metformin impairs the motility of oral cancer cells.

### Metformin restrains tumor growth and metastasis in xenograft model

**Figure 3:**
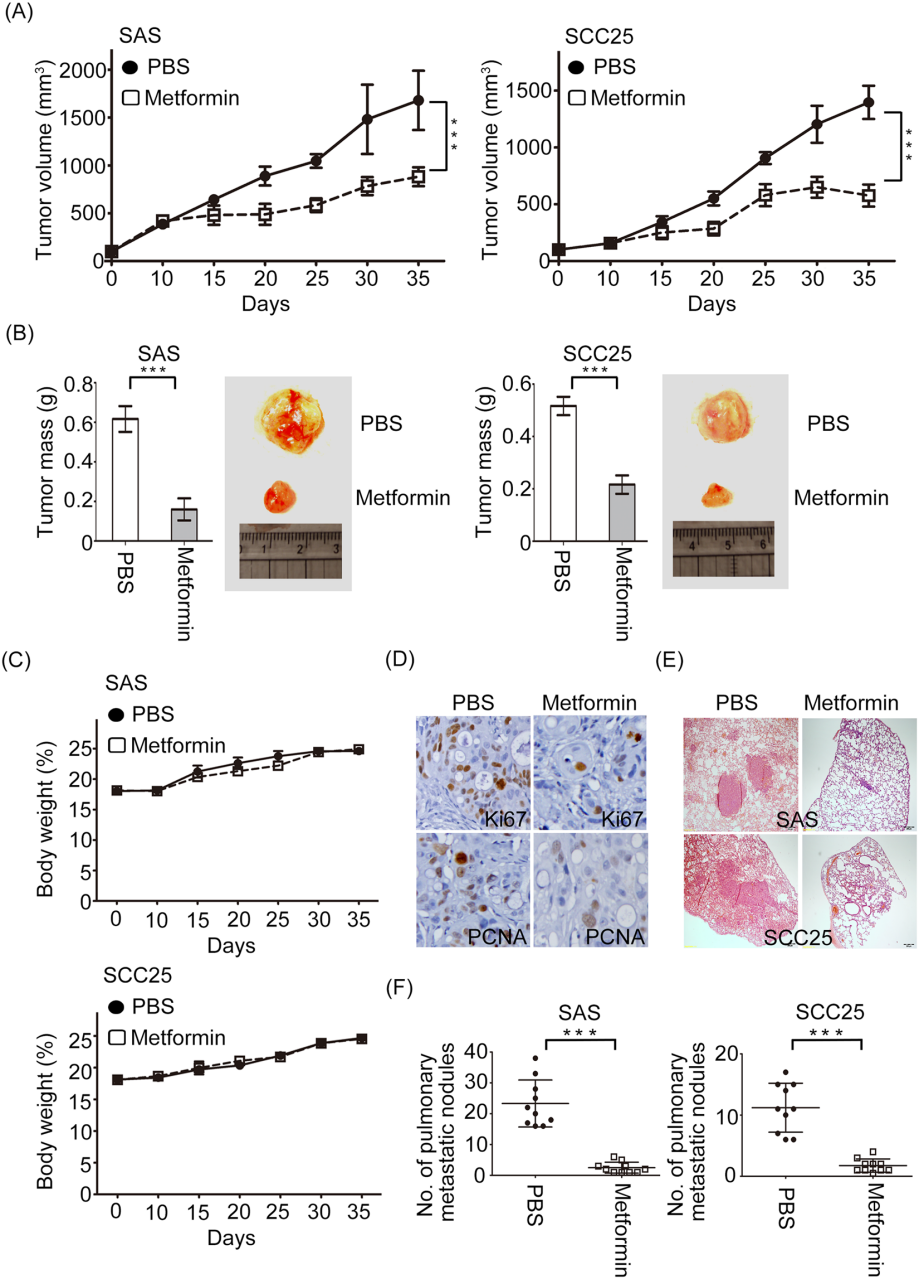
The effects of metformin on nude mice bearing SAS and SCC25 xenograft tumors. (**A**) Growth curves of xenograft tumors after the injection of SAS cells or SCC25 cells treated with PBS or metformin (5 mM) were measured. Tumor volume was evaluated at indicated times. (**B**) The effects of metformin on tumor mass. Tumor weight and representative tumors were measured and shown. (**C**) The body weights of mice were inspected during administration of metformin or control. (**D**) Representative images of expression levels of Ki-67 and PCNA in excised SAS-xenograft tumor tissues with metformin-treated mice or PBS group. (**E**) Representative images of lungs with HE-staining were isolated from mice that received tail vein injection of either SAS or SCC25 cells in the absence or presence of metformin treatment (5 mM). (**F**) The numbers of pulmonary metastatic nodules in the lung were counted and analyzed. All quantitative data are presented as mean ± SD. Data are represented as mean ± SD of three individual experiments. (***p < 0.01).

To further illustrate the anti-tumor activity of metformin *in vivo*, we assessed its effect in a xenograft model. SAS and SCC25 cell lines pretreated with PBS or metformin for 2 weeks were transplanted subcutaneously to the flank of nude mice. The tumor volumes and body weights of mice were measured every 5 days after tumor cells inoculated into the subcutaneous of nude mice. After 5 weeks, the tumor mass were dissected and measured from the mice. It was obvious that the tumor volume and mass from the metformin treated group was reduced significantly when compared with the control group. Moreover, the control group exhibited evidently faster growth rate (Fig. 3A and B). However, there was no significant weight loss and death of mice due to metformin toxicity, compared to control group (Fig. 3C). Immunohistochemical staining showed that inhibition of both PCNA and Ki67 expressions in excised tumor tissues of metformin-treated mice compared to control (Fig. 3D). Next, we studied the effect of metformin on tumor metastasis *in vivo*. SAS and SCC25 cells-treated with metformin or not were injected into nude mice via tail veins. After 35 days, lungs of the mice were dissected and performed histological examination by hematoxylin and eosin (H&E) staining. The results indicated that metformin-treated either SAS or SCC25 cells displayed a lower metastatic ability compared to control group (Fig. 3E). Moreover, the counts of pulmonary metastatic nodules showed significant reduction in the metformin-treated group (Fig. 3F). Altogether, metformin could suppress tumorigenesis and metastasis of oral cancer cells *in vivo*.

### Metformin down-regulates Aurora-A expression independent of AMPK in oral cancer cells

**Figure 4:**
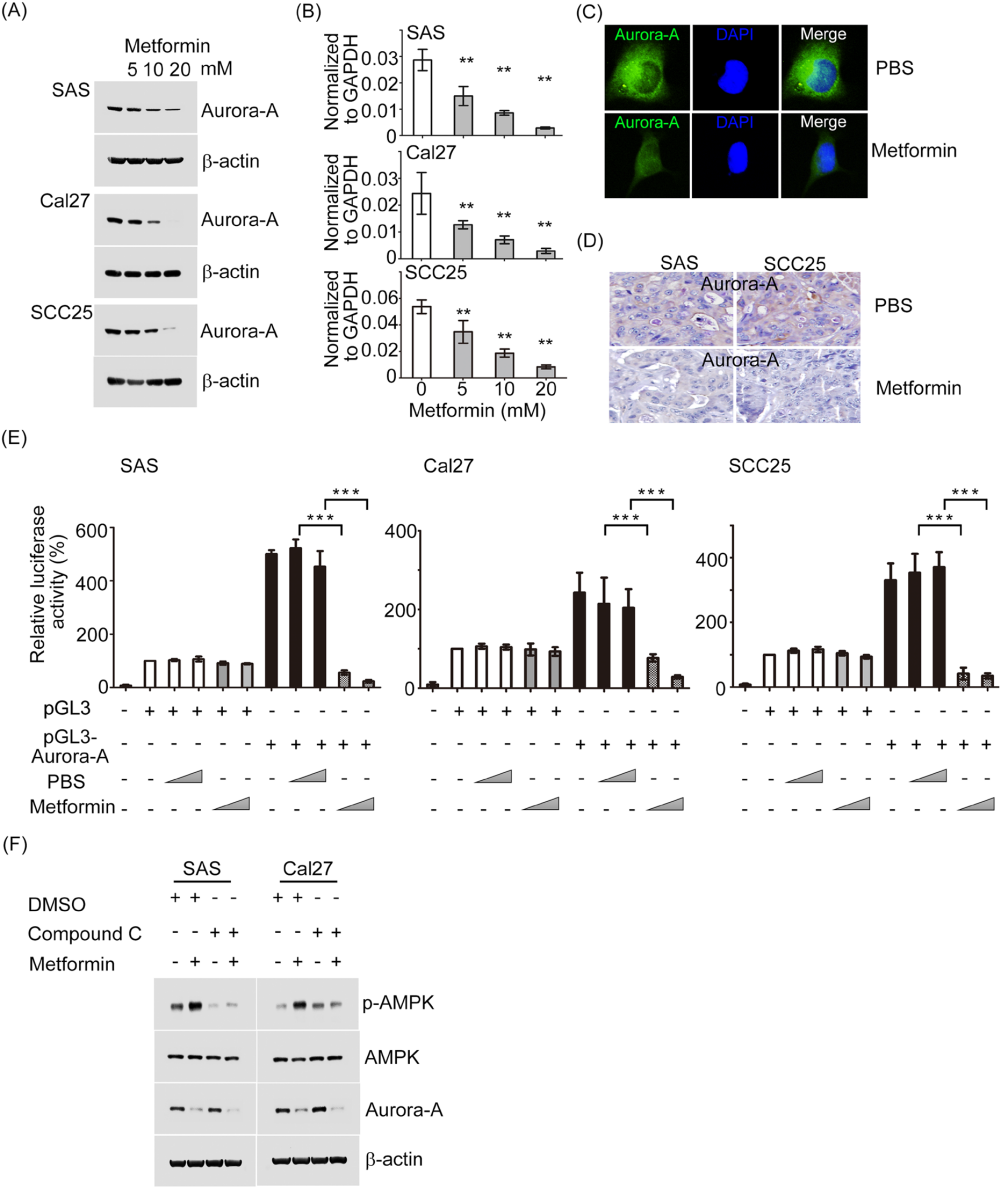
Metformin reduced Aurora-A expression levels *in vitro* and *in vivo*. (**A** and **B**) SAS, Cal27, and SCC25 cells were plated in 6 cm dish and treated with metformin as indicated concentrations or PBS for 48 hour. Following treatment, cells were harvested and the mRNA and protein expression profiles of Aurora-A in each sample were determined using western blot and Q-RT-PCR. (**C**) The expression and distribution of Aurora-A in oral cancer cell lines in the absence or presence of metformin was evaluated by immunofluorescence microscopy. (**D**) Representative images of immunohistochemistry of Aurora-A. (**E**) Relative luciferase activity is shown for reporter constructs containing Aurora-A promoter region. pGL3 or pGL3-Aurora-A promoter was co-transfected with 200 ng of internal control plasmid expressing Renilla-luciferase into the oral cancer cell lines. After 24 hour, cells were treated with in the absence or present of metformin in a dose-dependent manner. Relative luciferase activities were measured at 48 h after treatment. The relative luciferase activity was determined after normalizing to Renilla-luciferase activity. (**F**) SAS and Cal27 cells were pretreated for 1 h with 5 μM compound C (AMPK inhibitor) and then the cells were treated for an additional 24 h with 5 mM metformin. Phosphorylated AMPK, total AMPK, and Aurora-A protein expressions were assessed by Western blotting. All values represent the mean ± SD of three independent transfections. (**p < 0.01; ***p < 0.001).

We next explored the mechanism underlying the repressive role of metformin in oral cancer cell growth, migration and invasion. In oral cancer, the amplification and/or overexpression of Aurora-A occurs in up to 65% of tumor tissues and its activation could promote oral cancer cell proliferation and metastasis^18^.We recently reported that aberrant Aurora-A kinase expression/activity promotes the development of distant metastases originating from oral cavity squamous cell carcinomas^17^. To test if Aurora-A expression was regulated by metformin, the mRNA and protein levels of Aurora-A were examined by Q-RT-PCR and Western blotting. The results showed that the endogenous Aurora-A expression was decreased upon metformin treatment in a dose-dependent manner in SAS, Cal27, and SCC25 cells (Fig. 4A). Moreover, metformin could also suppress Aurora-A expression at the mRNA level (Fig. 4B). Analysis of the endogenous Aurora-A level in metformin-treated cell lines showed that it had 2–5-fold reduced of Aurora-A expression at RNA and protein levels, compared to the control group. Aurora-A protein expression was also confirmed in cells and xenograft-bearing tumor tissues of metformin treatment by immunofluorescence and immunohistochemistry (Fig. 4C and D). To verify if the transcriptional level of Aurora-A was regulated by metformin, a luciferase reporter containing the Aurora-A promoter was constructed. The reporter assay showed that metformin significantly decreased the luciferase activity of Aurora-A in the SAS, Cal27 and SCC25 cells in a dose-dependent manner (Fig. 4E). In order to determine if down-regulation of Aurora-A expression by metformin is dependent on AMPK pathway, we decided to inhibit AMPK activity by using compound C. We found that Compound C did not reverse the suppressive effects of metformin on Aurora-A expression (Fig. 4F). Taken together, these results illustrate that metformin represses Aurora-A expression in oral cancer cells by an alternative, AMPK-independent signaling mechanism.

### Aurora-A blockade with metformin controls growth and motility of tumor cells

**Figure 5:**
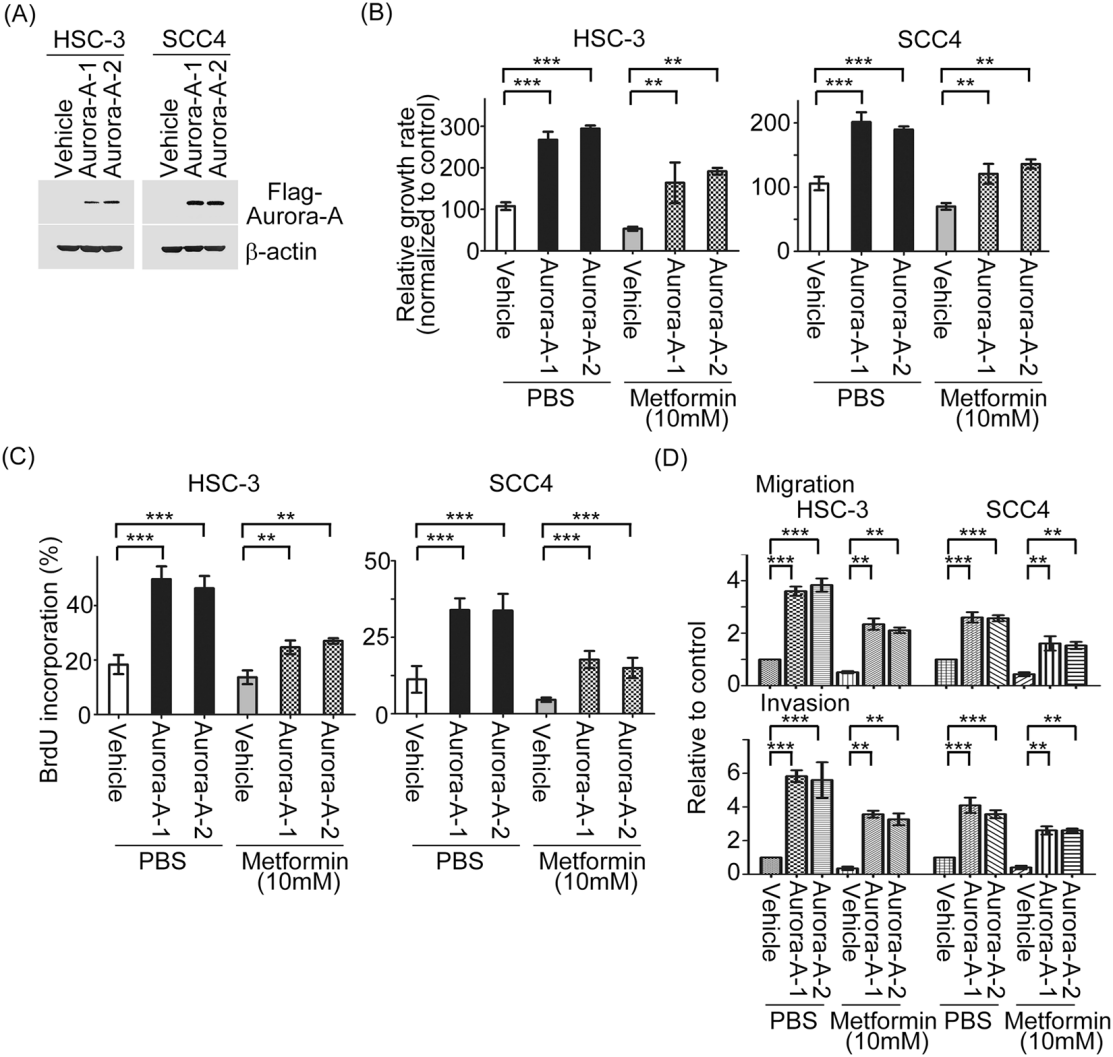
Aurora-A expression involves in Metformin-inhibited oral cancer cell proliferation, migration and invasion. (**A**) Q-RT-PCR and Western blot showing Aurora-A mRNA and protein levels in exogenous HSC-3/Aurora-A and SCC4/Aurora-A stable cells. (**B** and **C**) The abilities of cell growth and BrdU incorporation of Aurora-A stable cells vs vehicle groups in the absence or presence of metformin (10 mM) were examined. (**D**) The migratory and invasive abilities of Aurora-A stable cells vs control groups under metformin (10 mM) treatment in a dose-dependent manner were examined by using Transwell assay. Data are represented as mean ± SD of three individual experiments. (**p < 0.01; ***p < 0.001).

As describe above, metformin inhibited oral cancer cell growth, migration and invasion and decreased Aurora-A mRNA and protein expressions, we were interested in exploring if metformin-mediated the suppression of proliferation and metastasis were ascribable to Aurora-A expression. First, the Aurora-A stably expression cells in HSC-3 and SCC4 were established and confirmed (Fig. 5A). Next, Aurora-A transfectants and vehicle were treated with metformin and their viabilities were measured by MTT and BrdU assays. Interestingly, Aurora-A-overexpressing cells showed significantly resistant to metformin than that in vehicle control treated with metformin for 48 hour (Fig. 5B). Consistently, Aurora-A enhanced BudU incorporation in both Aurora-A/HSC-3 and Aurora-A/SCC4 transfectants in the present of metformin, compared to vehicle (Fig. 5C). Using the same cell panels, we also found that restoration of Aurora-A could reverse the metformin-mediated inhibition of migratory and invasive abilities in oral cancer cells (Fig. 5D). Taken together, these results suggest that metformin attenuates oral cancer cells proliferation and metastasis via Aurora-A expression.

### LSF regulates Aurora-A expression via association with Aurora-A promoter in oral cancer cells

**Figure 6:**
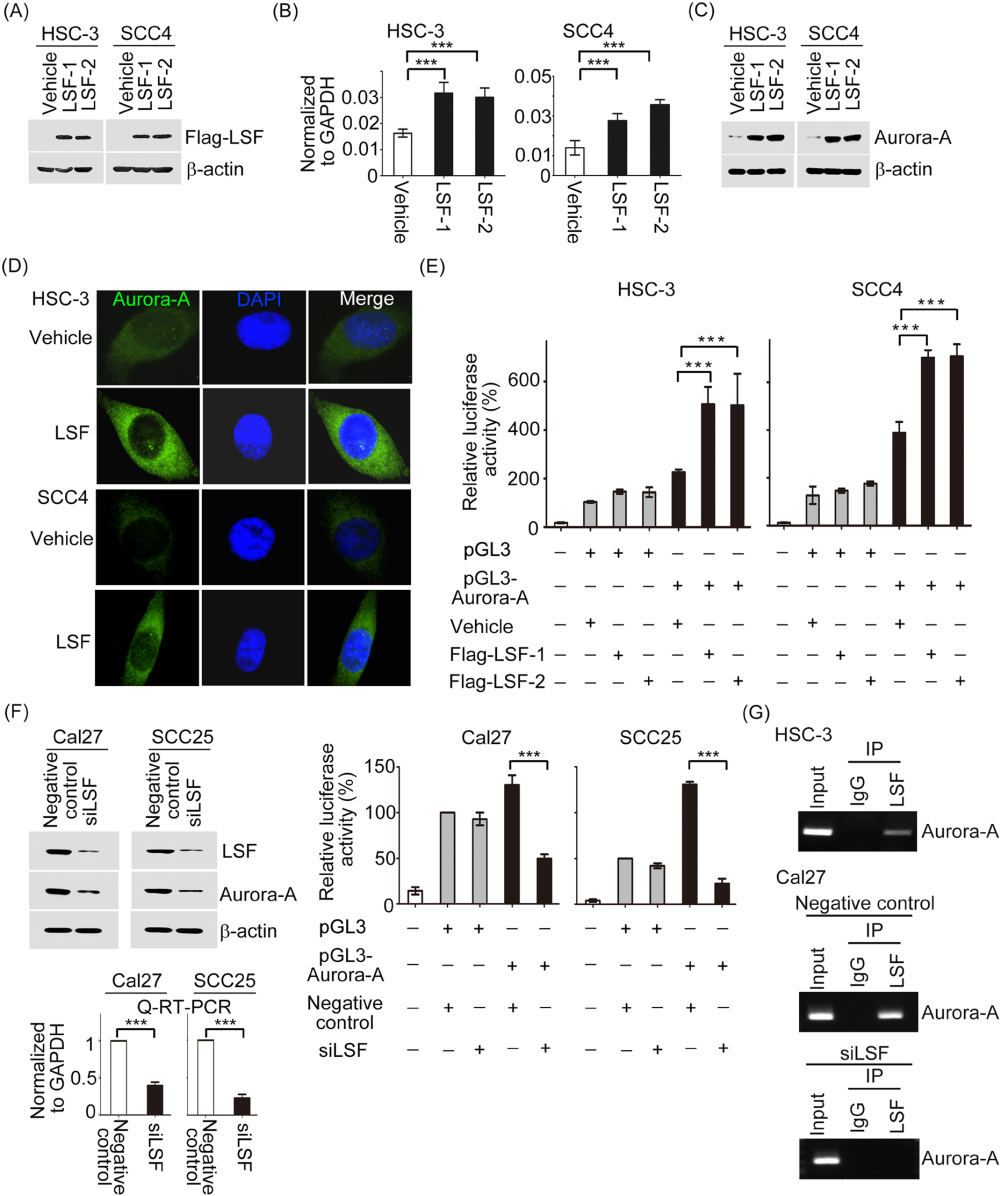
LSF regulates Aurora-A expression in transcriptional and post-transcriptional levels in oral cancer cells. (**A**) Q-RT-PCR and Western blot showing LSF mRNA and protein levels in exogenous HSC-3/LSF and SCC4/LSF stable cells. (**B** and **C**) The endogenous Aurora-A mRNA and protein expression profiles in gain-of-function of LSF oral cancer cells were determined by Q-RT-PCR and Western blotting. (**D**) The subcellular localization and expression level of endogenous Auorar-A protein in gain-of-function of LSF oral cancer cell lines. Cell nucleus appeared as blue fluorescence stained with DAPI. (**E**) LSF transfectants transfected with 200 ng of the pGL3 promoter luciferase construct containing the Aurora-A promoter regions, together with 200 ng of internal control plasmid expressing Renilla-luciferase. Relative luciferase activities were measured at 48 h after transfection. All experiments were performed at least twice, in triplicate. (**F**) The Aurora-A’s mRNA and protein expressions, and its luciferase activity were estimated in siLSF-Cal27 and siLSF-SCC25 cells by Western blotting, Q-RT-PCR and promoter assay. (**G**) ChIP analysis of the physical association between LSF and the Aurora-A promoter region. Crosslinked protein-chromatin complexes were immunoprecipitated from parental HSC-3 cells, or in Cal27 cells transfected with negative control or siLSF using an anti-LSF antibody or human IgG as a control. Error bars represent mean ± SD of three independent experiments. (***p < 0.001).

A growing body of evidence demonstrated that LSF plays oncogene or tumor suppressor role in variety of human cancers^19, 20^. Recent studies have highlighted that the LSF activation could be regulated by Prolyl isomerase, Pin1 whose biological functions in human cells were mediated by Aurora-A^21^. We hypothesized that Aurora-A and LSF may have functional links in oral cancer. To examine this hypothesis, LSF stably expression cells in HSC-3 and SCC4 were established and its protein expression were confirmed (Fig. 6A). Next, we performed Q-RT-PCR to determine effects of ectopic LSF on Aurora-A expression in both HSC-3 and SCC4 cells. As shown in Fig. 6B, ectopically expressed LSF increased Aurora-A mRNA levels remarkably. Concomitant with RNA results that increased Aurora-A protein level in LSF-overexpression HSC-3 and SCC4 cells were also observed (Fig. 6C). Furthermore, immunofluorescence experiments demonstrated that Aurora-A protein levels were also increased upon expression of LSF (Fig. 6D). We next assayed effects of LSF on transcriptional activity at the Aurora-A promoter. Aurora-A promoter plasmid were transfected into LSF stable cells and the promoter activity was determined. As expect, Aurora-A luciferase activity was increased by enforced LSF expression in LSF-HSC-3 and LSF-SCC4 stable cells but not by vehicle groups (Fig. 6E). To confirm these findings, we introduced siRNA targeting LSF or a negative control into Cal27 and SCC25 cells. We observed that the mRNA and protein expression levels and luciferase activity of Aurora-A were suppressed while endogenous LSF was abrogated by LSF-mediated siRNA (Fig. 6F). To gain deeper mechanistic insights into the LSF in modulating *Aurora-A* promoter activity, we pursued our investigation by conducting chromatin immunoprecipitation (ChIP) assay. The data illustrated that LSF bound to the *Aurora-A* promoter *in vivo* (Fig. 6G). However, LSF bound to the *Aurora-A* promoter much less efficiently in LSF-knocking down cells, suggesting that the association was specific (Fig. 6G). Taken together, this data identifies Aurora-A as a direct downstream target of LSF.

### Metformin inhibits aggressive phenotype of oral cancer cells is dependent on LSF

**Figure 7:**
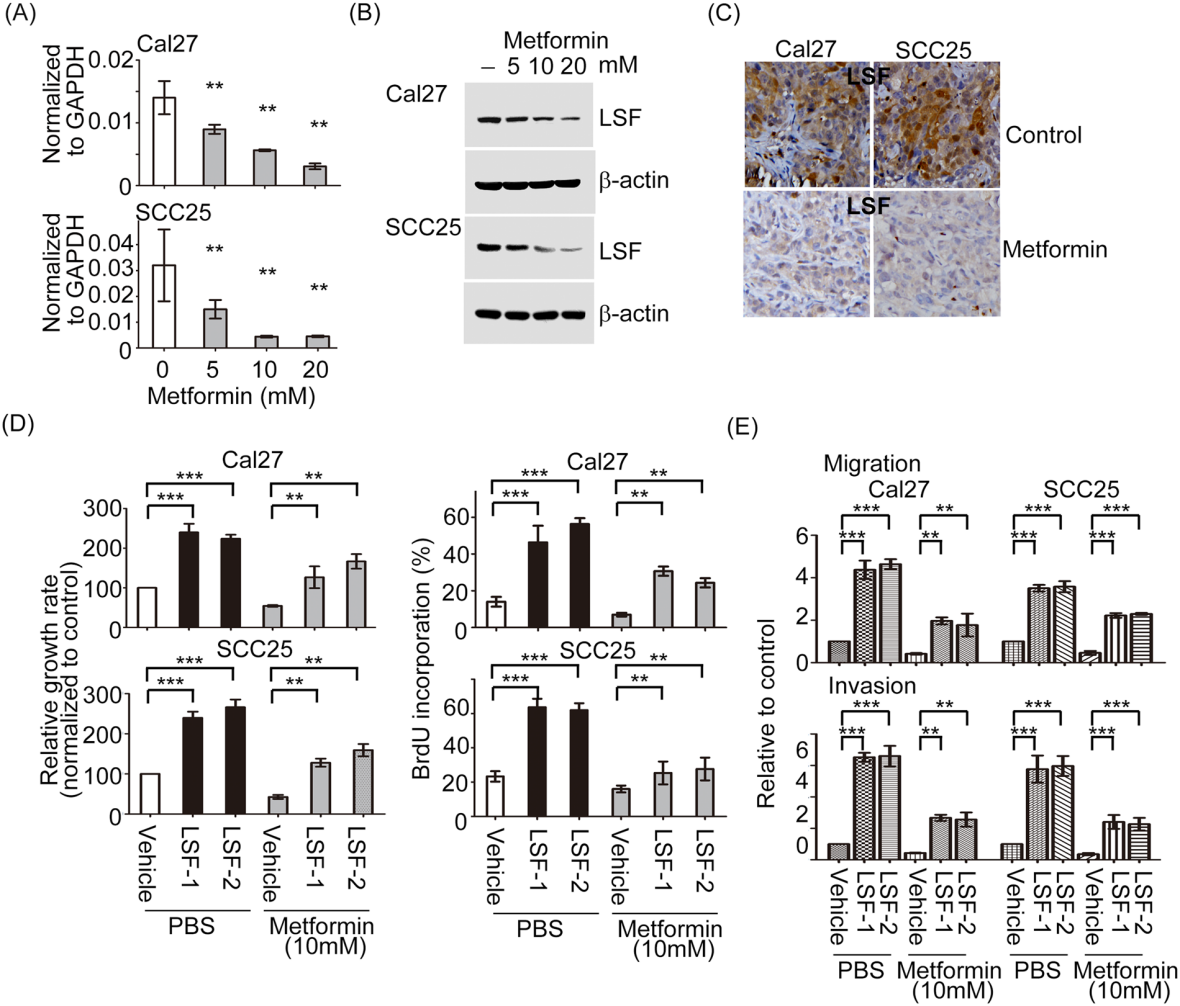
Metformin suppresses LSF-elicited cell growth, migration and invasion in oral cancer cells. (**A** and **B**) Cal27, and SCC25 cells were treated with metformin for 48 hour. Following treatment, cells were harvested and the mRNA and protein expression profiles of LSF in each sample were determined using Western blotting and Q-RT-PCR. (**C**) Representative images of immunohistochemistry of LSF. (**D**) The abilities of cell growth and BrdU incorporation of LSF stable cells vs vehicle groups in the absence or presence of metformin for 48 hour were examined. (**E**) The migratory and invasive abilities of LSF stable cells vs control groups under metformin treatment were examined by using Transwell assay. All values represent the mean ± SD of three independent transfections. (**p < 0.01; ***p < 0.001).

To further validate the idea that the influence of metformin on tumor formation and motility dependent on LSF, we first intended to examine whether endogenous LSF expression was modulated by metformin. As shown in Fig. 7A and B, notably, the mRNA and protein expression levels of LSF were reduced in a dose-dependent manner upon metformin treatment in both Cal27 and SCC25 oral cancer cells. Tumor sections obtained from mice injected with metformin were further subjected to immunohistochemical analysis. The results showed that tumor treated with metformin resulted in downregulation of LSF (Fig. 7C). To test whether metformin-induced inhibition of cell viability, migration and invasion could be reversed by restoration of LSF expression, the LSF stable clones under metformin treatment were applied. Re-expression of LSF significantly reversed metformin-mediated inhibition of cell growth and BrdU incorporation in both Cal27 and HSC-2 oral cancer cells (Fig. 7D). Additionally, Transwell assays were performed to test whether increased LSF affected migration and invasion of oral cancer cells treated or not with metformin. Importantly, we found that metformin severely decreased the abilities of cell migration and invasion were dramatic reversed in reinforced LSF expression in oral cancer cells (Fig. 7E). We conclude that LSF is required for metformin-mediated proliferation, migration, and invasion of oral cancer cells.

### Correlation of LSF and Aurora-A expression with oral cancer progression

**Figure 8:**
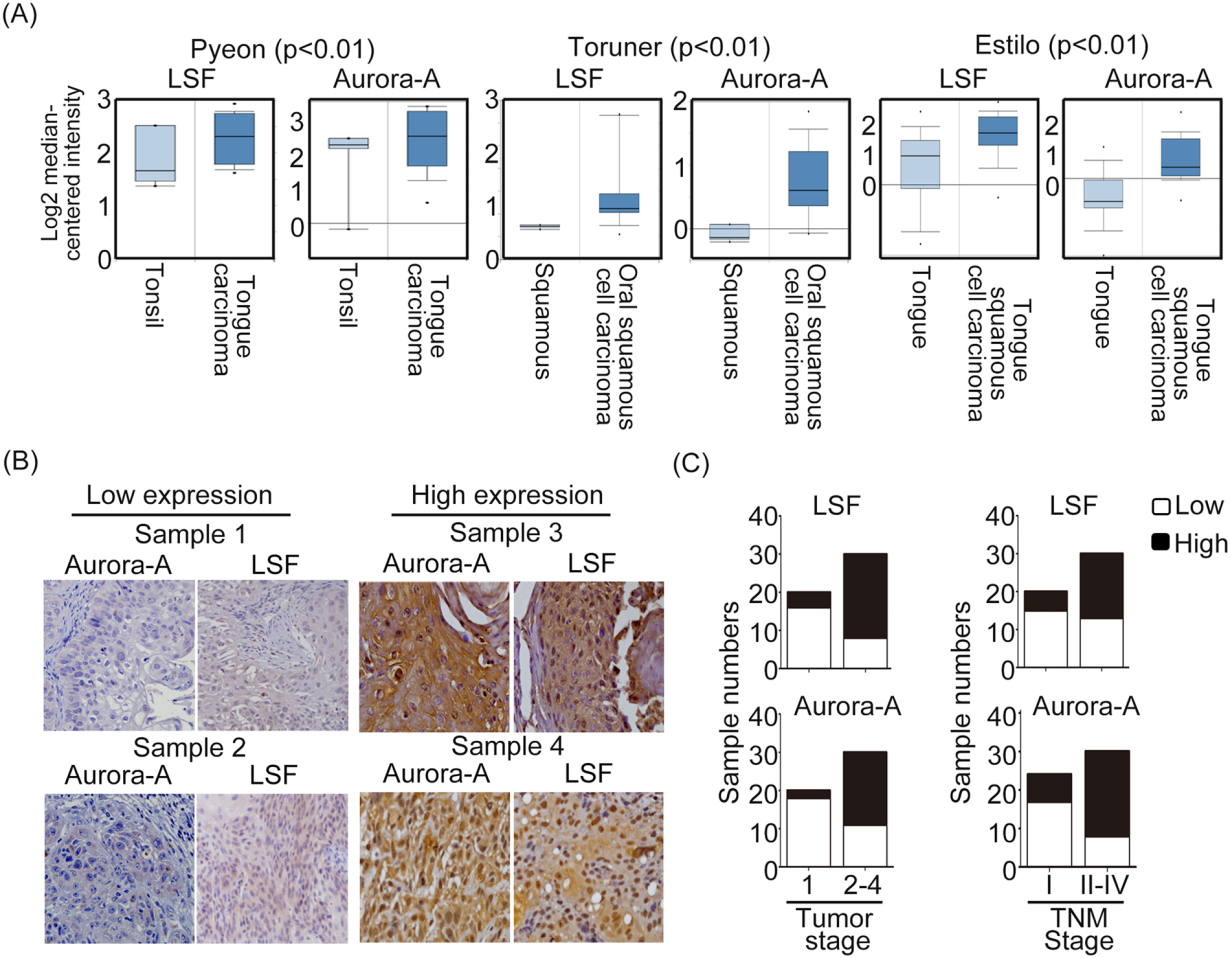
LSF expression positively correlated with Aurora-A expression in human oral cancer specimens. (**A**) Box plots representing Aurora-A and LSF expressions from oral tumor samples at the same databases. Oncomine was used for analysis and visualization. (**B**) Immunohistochemical staining of Aurora-A and LSF in oral cancer tissues showed that Aurora-A expression was positively correlated with LSF expression. (**C**) Statistical analysis showing that LSF expression levels in oral cancer specimens was significantly correlate with tumor and TNM stages.

According to above results prompted us to analyze the expressions of LSF and Aurora-A in oral cancer clinical specimens. Analysis of publicly accessory gene expression database using the Oncomine revealed that expression of both LSF and Aurora-A was significantly increased in oral cancer tissues than that in normal tissues, implicating a clear correlation between up-regulation of LSF and Aurora-A and oral cancer progression (Fig. 8A). We next measured the expression of LSF and Aurora-A in a cohort of oral cancer samples. Immunohistochemistry assays revealed that there was a significant positive correlation between LSF and Aurora-A expression in the 50 oral cancer samples (Fig. 8B). On the basis of the clinical staging, advanced tumor size and TNM stage had higher LSF and Aurora-A expression profiles in compared with the early tumor size and TNM stage of patients (Fig. 8C). The correlation between each paired IHC scores of LSF and Aurora-A were analyzed by Spearman’s rank tests. It showed that there were positive correlations between LSF and Aurora-A (rho = 0.688, p < 0.001) (Table 1). In addition, χ^2^ test was done to assess the association between LSF and Aurora-A. LSF again is significantly associated with Aurora-A (p < 0.001). Taken together, these results indicated that LSF expression is significant positively correlated with Aurora-A in human oral cancer specimens.

**Table 1:** The correlation between LSF, and Aurora-A expression in oral cancer specimens.

	LSF	Aurora-A
**LSF**		
Spearman’s rank correlation	1	
Sig. (2-tailed)	.	
Numbers	50	
**Aurora-A**		
Spearman’s rank correlation	0.688**	1
Sig. (2-tailed)	<0.001	.
Numbers	50	50

*Statistically significant (p < 0.05).

## Discussion

In this study, we showed that metformin not only suppressed oral cancer cell growth and metastasis, but also inhibits tumorigenesis in a xenograft model. Here, we also identified a novel mechanism that metformin-suppressed oral cancer cell development was correlated with a decrease of expression of Aurora-A, a promising prognostic biomarker. In addition, we documented that Aurora-A mediated malignancy phenotype of oral cancer was modulated by LSF. In a cohort of 50 oral cancer patients with paired samples, Aurora-A expression was positively correlated with LSF expression. Moreover, our data suggested that LSF/Aurora-A signaling could be intracellular target of the metformin-mediated anti-tumor development in oral cancer cells. This study is the first report to connect the LSF and Aurora-A synergistically to participate in the carcinogenesis process of oral cancer.

It has been well-established that metformin exhibits pleiotropic mechanisms for cell protection in different signaling pathways, including AMPK-dependent or AMPK-independent such as TGF-β/IL-6^22^, Wnt/β-catenin^23^; autophagy pathway^24^, and COX2/PGE2/STAT3^25^. The AMPK-dependent mechanism of metformin action was established by Zhou in 2001^26^. The results demonstrated that metformin promotes AMPK activation and used the compound C, a AMPK inhibitor to indicate that AMPK required for the drug’s inhibitory effect on glucose production in primary hepatocyte. Later, Shaw *et al*. found that LKBl, an upstream activator of AMPK, activated by metformin could alter the cell’s gluconeogenic programme via inhibition of cAMP response element-binding protein (CREB). A growing body of evidence showed that a downstream of AMPK, acetyl-CoA carboxylase (ACC), a precursor of lipogenesis, not only participated in metformin-induced improvements in insulin action in mice, but also modulated metformin-suppressed the proliferation rate of cancer cells^27^. Aside from the metabolic diseases, AMPK activation by metformin may regulate cell cycle progression. For instance, AMPK diminished the c-Myc expression via increasing miR-33a expression^28^. Additionally, AMPK phosphorylated p53 at Ser15 resulted in cell cycle arrest^29^.

AMPK-independent mechanisms have been reported to demonstrate the anti-cancer properties of metformin. Metformin can prevent DNA damage and mutation by inhibiting ROS generation by complex I^30^. In prostate cancer, REDD1, a negative regulator of mammalian target of rapamycin (mTOR), is regulated by p53 and is required for metformin effects on cell-cycle arrest^31^. Inactivation of Stat3-Bcl-2 pathway contributes to metformin-induced growth inhibition of esophageal squamous cell carcinoma (ESCC) by facilitating crosstalk between apoptosis and autophagy^32^. About glucose metabolism in cancer cells, metformin could decrease ^18^F-fluorodeoxyglycose uptake by directly inhibiting enzymatic activity of HKII and HKI and mimicking G6P in human non-small cell lung cancer cells^33^.

Numerous preclinical and clinical studies have indicated anti-tumor effects of metformin, conducing to an explosion of interest in evaluating this agent in human cancer. Most *in vitro* studies using concentrations of metformin (1–40 mM), which is above the practical therapeutic plasma levels (2.8–15 μM) in humans^34^. With regard to the issue of inconsistency that the metformin concentrations used between in cultured cells and in patients, a possible explanation is that the growth circumstance of tumor cells is discrepancy. The cancer cell lines are often maintained in non-physiological conditions with high concentrations of growth factors, glucose and hormones that may account for the elevated doses of metformin required to elicit cellular responses *in vitro* ^35^.

Aurora-A, is frequently overexpressed in a variety of human cancers, such as head and neck cancer, ovarian cancer, colorectal cancer, breast cancer, indicating that Aurora-A plays critical role in tumorigenesis^36–39^. A growing body of evidence indicated that Aurora-A represent a promising diagnostic or prognostic marker. Our previous report indicated that increased Aurora-A expression was significantly associated with poor disease-free and overall survival of patients with head and neck squamous cell carcinoma^16^. Goos *et al*. showed that Aurora-A overexpression in liver metastatic lesions compared to corresponding primary tumors was associated with poor clinical outcome^40^. *In vitro* study, Aurora-A could regulate many oncoproteins or tumor suppressor proteins to involve in cancer progression. Aurora-A activated AKT/mTOR signaling via phosphorylating AKT oncoproteins which is essential to promote U2OS tumorigenicity^41^. Aurora-A dephosphorylated cofilin and activated cofilin-F-actin pathway for actin reorganization and polymerization to accelerate breast cancer metastases^42^. Rap1 activation was required for Aurora-A regulation to mediate distant metastases originating from oral cavity squamous cell carcinomas^17^. Here, for the first time, we showed that metformin restrained oral cancer cell growth and metastasis via suppression of Aurora-A expression. Highly expression of Aurora-A in oral cancer cells led to the resistance to metformin. Our finding documented that metformin downregulates Aurora-A at least in part by altering Aurora-A’s transcriptional and post-transcriptional levels represents a new mechanism of metformin.

LSF, a transcriptional factor, plays pivotal role for cell cycle progression, DNA synthesis, and cancer cell survival. Highly expression of LSF might enlarge all of these effects, thus promoting transformation of cancer cells^43^. However, the relationship between LSF signaling and its biological in oral cancer are speculative at present. Our results suggest that LSF participates in dyregulated Aurora-A signaling in tumorigenesis of oral cancer. Constitutively active LSF increased Aurora-A expression, which promoted cancer cell growth, migration and invasion in oral cancer cells. Conversely, inhibition of endogenous LSF by LSF-mediated siRNA suppressed the transcriptional and post-transcriptional levels of Aurora-A. In a cohort of paired oral cancer specimens, LSF protein expression profile was positively correlated with Aurora-A expression. Hence, our contribution to the field is identification of LSF as a “missing link” between Aurora-A and tumor cell growth and motility.

In conclusion, LSF is novel mediator of Aurora-A signaling and plays a pivotal role in the development and progression of human oral cancer. Importantly, metformin could block LSF/Aurora-A signaling, leading to malignancy phenotype of oral cancer. These findings show that metformin is promising in the treatment of OSCC although more prospective clinical trials are necessary to confirm our findings.

## Materials and Methods

### Human oral cancer tissues and IHC

Commercially purchased tissue microarrays (TMAs) include 50 samples of oral cancer (US Biomax, Inc., Rockville, MD, USA; catalog number HN802). This study was approved by the Medical Ethics and Human Clinical Trial Committee at Chang Gung Memorial Hospital. Tissues were fixed with 10% buffered formalin embedded in paraffin and decalcified in 10% EDTA solution. Representative blocks of the formalin-fixed, paraffin-embedded tissues were cut to 4 mm and deparaffinized with xylene and rehydrated in a series of ethanol washes (100, 90, 80, and 70%). Slides were washed with phosphate-buffered saline (PBS) and treated with 3% H_2_O_2_ for 30 minutes to block endogenous peroxidase activity. Next, the sections were microwaved in 10 mM citrate buffer, pH 6.0, to unmask the epitopes. After antigen retrieval, the sections were incubated with diluted anti-Aurora-A, and anti-LSF antibodies for 1 h followed by washing with PBS. Horseradish peroxidase/Fab polymer conjugate (PicTure^™^-Plus kit; Zymed, South San Francisco, CA, USA) was then applied to the sections for 30 min followed by washing with PBS. Finally, the sections were incubated with diaminobenzidine for 5 min to develop the signals. The reactivity level of the immunostained tissues was evaluated independently by two pathologists who were blind to the subjects’ clinical information. Between 15 and 20 high-power fields were viewed. Criteria were developed for quantitating the immunoreactivities of the Aurora-A, and LSF stainings in tumor sections using a score range of 0 to +3, where 0 indicated no positive cell staining, +1 less than 10% positive cell staining, +2 10–30% positive cell staining, and +3 more than 30% positive cell staining. Similarly, the stain intensity was graded as +0, +1, +2, or +3 as previously described^16^.

### Cell culture, transient transfection, the establishment of stable clones, and luciferase assay

Cell lines were obtained from the American Type Culture Collection. All cell culture-related reagents were purchased from Gibco-BRL (Grand Island, NY, USA). All cells were grown in DMEM containing 10% FBS and 100 U/ml penicillin and streptomycin (Gibco-BRL). Flag-vector, Flag-Aurora-A and Flag-LSF were transiently transfected into cancer cells using Lipofectamine (Invitrogen) according to the manufacturer’s instructions. Mixed-steady expressing Aurora-A or LSF were selected with 400 μg/ml G418 (Calbiochem Novabiochem, San Diego, CA, USA) for two weeks. The cell were then harvested and analyzed for exogenous Aurora-A and LSF expressions by Western blotting. 5′-upstream fragments of *Aurora-A* gene (−1~−2000) was amplified from human genomic DNA and verified by sequencing. The PCR fragments were cloned into firefly luciferase reporter vector pGL3-Basic (Promega) NheI and HindIII sites which were designed into the forward and the reverse primers, respectively. For co-transfection experiments, cells were co-transfected with 100 ng firefly luciferase reporter plasmids, and 10 ng of pRL-TK *Renilla* luciferase internal control plasmid. After 24 h, the luciferase activity was measured using Dual Glo™ Luciferase Assay System (Promega). One double-stranded synthetic RNA oligomers (5′-CGUCCAAGGUUAACCAUUUtt-3′ deduced from human *LSF*, and one negative control siRNA (#4611 G; Ambion) were used in the siRNA experiments.

### Immunoblot analysis

Samples were homogenized in RIPA lysis buffer (50 mM Tris-HCl, pH 7.5, 150 mM NaCl, 1% NP-40, 0.5% Na-deoxycholate, and 0.1% SDS). The protein concentration in each sample was estimated by Bio-Rad Protein Assay (Bio-Rad, Hercules, CA, USA). Immunoblotting was performed according to standard procedures. Antibodies used in this study include Aurora-A (monoclonal; Epitomics, Burlingame, CA, USA), LSF (monoclonal; Millipore), and β-actin (monoclonal; Santa Cruz Biotechnology, Santa Cruz, CA, USA). The first antibodies were detected by incubation with secondary antibodies conjugated to HRP (Bio/Can Scientific, Mississauga, ON, Canada) and developed using Western Lighting Reagent.

### Indirect immunofluorescence analysis and Time lapse microscopy

The indirect immunofluorescence staining on the cells treated with metformin or LSF stable cells was performed with anti-Aurora-A, at RT for 2 h. The sections were then washed three times with PBST and incubated with DAPI and goat-anti-rabbit-FITC (Jackson, ImmunoResearch) at RT for 1 h. After washing with PBST, the sections were mounted with GEL/Mount (Biomeda corp, Foster, CA). The fluorescence images on the slips were examined using a confocal microscope (Olympus FV10i). SAS cells were seeded on 4 well chambered cover-glass for 24 hours and treated with either PBS or 10 mM metformin. The chamber was mounted onto the stage of an inverted microscope maintaining normal growth condition with incubation system. Confocal images of the cells were acquired by a spinning disk confocal system every 30 min. Imaging data were analyzed using MetaMorph (Molecular Devices, Sunnyvale, CA).

### RNA extraction, and quantitative RT-PCR

Samples were frozen in liquid nitrogen and stored at −80 °C prior to RNA extraction. The cells were homogenized using a Mixer Mill Homogenizer (Qiagen, Crawley, West Sussex, UK). Total RNA was prepared from the frozen tissue samples using an RNeasy Mini Kit (Qiagen) according to the manufacturer’s instructions. The RNA (2 μg) was then reverse transcribed into cDNA using SuperScript II Reverse Transcriptase (Invitrogen, Carlsbad, CA, USA). For Q-RT-PCR, *Aurora-A* and *LSF* Taq-Man probe (ABI) were used to perform the study. Data were represented as mean ± s.d. To analyze the distribution of control and experimental groups, we performed the Wilcoxon signed rank test between two groups for statistical analysis. A *P*-value of less than 0.05 was significant. *GAPDH* (ABI) was used as an internal control for comparison and normalization the data. Assays were performed in triplicate using Applied Biosystems Model 7700 instruments.

### Cell viability assay and colony formation assay Flow cytometry analysis of the cell cycle

Cells (1 × 10^4^) in 200 μl medium were seeded in 96-well plates. Next day, the medium in each well was replaced with medium containing different concentrations of metformin and incubated for 48 h. Cell viability was then determined using the MTT assay. The plates were stored at 37 °C for 4 hour, and then 100 μL DMSO buffer was added and incubated in the dark for 10 min. Absorbance was measured on a microplate reader at 540 nm. The OD values were normalized with the value of control group. For colony formation assay, cells were seeded in 60-mm dishes at a density of 5 × 10^3^ cells. Next day, cells were treated with metformin (10 mM) for 20 days. Subsequently, cell colonies were counted after staining with 0.01% crystal violet. 1 × 10^5^ cells/well were cultured in 6-well plates and incubated overnight. The cells were then treated with 10 mM metformin for 48 h. Harvested cells were incubated with RNase for 30 min at 37 °C and 5 μl propidium iodide (PI) was added, followed by a 10 min incubation in the dark. The samples were subsequently analyzed using flow cytometry and the percentage of cells in each phase was determined using Kaluza software.

### Chromatin immunoprecipitation (ChIP)

ChIP assays were performed according to the protocol from Millipore (EZ–Magna ChIP G Chromatin Immunoprecipitation Kit, Millipore). Chromatin was precipitated using anti-LSF antibody and protein A agarose at 4 °C overnight and immune complexes were collected by centrifugation. Normal human IgG was used as a control. Cross-links were then reversed at 65 °C overnight. The purified DNA was amplified by PCR using Aurora-A promoter primers pre-denaturation for 3 min at 94 °C, denaturation at 94 °C for 20 sec., annealing at 47 °C for 30 sec., and extension at 72 °C for 30 sec. for a total of 30 cycles).

### Migration, invasion and wound-healing assays

Migration and invasion assays were conducted with cells in the absence or presence of metformin using 24-well Transwell chambers as described previous report^44, 45^ (8-µm pore size polycarbonate membrane; Costar, Corning, NY). For the migration (5 × 10^3^) and invasion (1 × 10^4^) assays, cells were suspended in 400 µl of DMEM containing 10% FBS, then seeded into the upper chamber; 600 µl of DMEM containing 10% FBS were added to the outside of the chamber. After being cultured at 37 °C under 5% CO_2_/95% air for 24 h, the cells on the upper surface of the membrane were removed with a cotton-tipped applicator and the migratory cells on the lower membrane surface were fixed with methanol and stained with Giemsa (Sigma, USA). Cell migration was evaluated by counting the number of cells that had migrated by 200X phase-contrast microscopy on three independent membranes, then normalized against the vehicle cells to determine the relative ratio. For the invasion assays, 80 µg/ml of Matrigel (BD Biosciences) were added to the upper surface of the membrane and allowed to gel at 37 °C overnight. A total cells (1 × 10^5^) in 400 µl of DMEM containing 10% FBS were seeded into the upper chamber, while 600 µl of DMEM containing 10% FBS were added to the outside of the chamber. The rest of the protocol was the same as that for the migration assays. For wound healing assay, Cells were initially seeded uniformly onto 60-mm culture plates with an artificial “wound” carefully created at 0 h. A P-10 pipette tip was used to scratch the sub-confluent cell monolayer. Micro-photographs were taken at 0 and 24 h. Quantitative analysis of the percentage of wound healing was calculated using the distance across the wound at 0 and 24 h, divided by the distance measured at 0 h for each cell line.

### Animal experiments and immunohistochemistry

Parental cells treated with or without metformin (5 mM) for 2 weeks were harvested, washed in PBS, and suspended in a mixture of PBS and Matrigel (BD Biosciences, San Jose, CA, USA). 1 × 10^6^ pre-treated cells were injected into the flanks of male nude mice. All animal experiments were carried out in accordance with protocols approved by the Animal Use and Management Committee of Kaohsiung Chang-Gung Memorial Hospital and E-DA Cancer Hospital. Mice were monitored daily and tumor volumes and body weights were measured twice weekly. To produce experimental lung metastasis, 5 × 10^5^ cells treated with or without metformin were injected into the tail veins of 6 weeks-old female nude mice. After 3 weeks, all the mice were killed under anesthesia. The lungs were collected and fixed in 10% formalin. At the completion of the study, tumors were excised, formalin-fixed and paraffin-embedded for immunohistochemical analysis.

### Statistical Analysis

Chi square test was used to evaluate the differences between the variables. A *p*-value less than 0.05 was considered to be significant in all of the statistical analyses.

## Declarations

### Ethics approval and consent to participate

This study was approved by the Animal Use and Management Committee at Chang Gung Memorial Hospital and E-DA Cancer Hospital.

### Availability of data and ﻿materials

The datasets supporting the conclusions of this article are included within the article.

## Electronic supplementary material

Supplementary Information

SAS control

SAS metformin
