# Supplementary material for: Metformin disrupts malignant behavior of oral squamous cell carcinoma via a novel signaling involving Late SV40 factor/Aurora-A
Source: Sci Rep. 2017 May 2;7:1358. doi: 10.1038/s41598-017-01353-8 (PMC5430965; doi:10.1038/s41598-017-01353-8)
Supplement: Supplementary file 1 — Supplementary Information [file 41598_2017_1353_MOESM1_ESM.doc]

**Title:** **Metformin disrupts malignant behavior of oral squamous cell carcinoma via a novel signaling involving Late SV40 factor/Aurora-A**

Chang-Han Chen^1,2,3^, Hsin-Ting Tsai^1,4^, Hui-Ching Chuang^4^, Li-Yen Shiu^5,6^, Li-Jen Su^7^, Tai-Jan Chiu^8^, Sheng-Dean Luo^4^, Fu-Min Fang^9^, Chao-Cheng Huang^10^, and Chih-Yen Chien^4#^

^1^Institute for Translational Research in Biomedicine, Kaohsiung Chang Gung Memorial Hospital, Kaohsiung, Taiwan

^2^Department of Applied Chemistry, and Graduate Institute of Biomedicine and Biomedical Technology, National Chi Nan University, Taiwan

^3^Center for Infectious Disease and Cancer Research, Kaohsiung Medical University, Kaohsiung, Taiwan

^4^Department of Otolaryngology, Kaohsiung Chang Gung Memorial Hospital, and Chang Gung University College of Medicine, Kaohsiung, Taiwan

^5^Department of Medical Research, E-Da Hospital, I-SHOW University, Kaohsiung, Taiwan

^6^Cell Therapy and Research Center, Department of Medical Research, E-Da Cancer Hospital, Kaohsiung, Taiwan

^7^Graduate Institute of Systems Biology and Bioinformatics, National Central University, Jhongli Taiwan

^8^Departments of Hematology-Oncology, Chang Gung University College of Medicine, Kaohsiung, Taiwan

^9^Department of Radiation Oncology, Kaohsiung Chang Gung Memorial Hospital and Chang Gung University College of Medicine, Kaohsiung, Taiwan

^10^Department of Pathology, Kaohsiung Chang Gung Memorial Hospital and Chang Gung University College of Medicine, Kaohsiung, Taiwan

**Supplementary information 1. The cells treated with metformin.** (A) SAS treated with PBS. (B) SAS treated with metformin.
